# XPG is a novel biomarker of clinical outcome in advanced non-small-cell lung cancer

**DOI:** 10.12669/pjms.293.3664

**Published:** 2013

**Authors:** Yi Yuli, Sun Zhe, WANG Xia, LI Siqing, WU Zhenxuan, ZHU Yu-hua, Sun Bing, Cui Jun-wei

**Affiliations:** 1 Yi Yuli, Nursing College of Nanchang University, Nanchang, China.; 2 Sun Zhe, Department of Oncology, the First Affiliated Hospital of Nanchang University, Nanchang, China.; 3 WANG Xia, The Fourth Department of Tuberculosis, The First Affiliated Hospital, Xinxiang Medical College, Weihui, China.; 4 LI Si-qing, The Second Department of Tuberculosis, The First Affiliated Hospital, Xinxiang Medical College, Weihui, China.; 5 WU Zhen-xuan, The Fourth Department of Tuberculosis, The First Affiliated Hospital, Xinxiang Medical College, Weihui, China.; 6 ZHU Yu-hua, The Fourth Department of Tuberculosis, The First Affiliated Hospital, Xinxiang Medical College, Weihui, China.; 7 Sun Bing, The Second Department of Tuberculosis, The First Affiliated Hospital, Xinxiang Medical College, Weihui, China.; 8 Cui Jun-wei, The First Department of Tuberculosis, The First Affiliated Hospital, Xinxiang Medical College, Weihui, China.

**Keywords:** Non-small-cell lung cancer, Xeroderma pigmentosum group G, Single Nucleotide Polymorphism, Progression-free survival, Overall survival

## Abstract

***Objective:*** Polymorphisms in XPG were considered to contribute to the clinical outcome of patients receiving platinum drug chemotherapy. We investigated the impact of several potential SNPs of XPG on the efficacy of platinum-based chemotherapy in NSCLC patients.

***Methods:*** A total of 433 patients were consecutively selected between Nov. 2006 and Dec. 2007, and were followed-up up to Nov. 2011. The genotyping of six SNPs (rs2296147, rs751402, rs873601, rs4150375, rs17655 and rs2094258) were genotyped using the Taqman real-time PCR method with a 7900 HT sequence detector system.

***Results:*** Patients carrying CT+TT genotype of rs2296147 had a significantly longer median PFS (17.5 months) and OS (26.8 months) than CC genotype. Hazard ratio (HR) for PFS and OS in patients with CT+TT genotype of rs2296147 was respectively 0.73(0.51-0.97) and 0.66(0.48-0.99) when compare CC genotype, respectively. Similarly, patients with rs2094258 AG+GG genotype had a longer median progression time (18.4 months) and overall survival time (27.3 months) when compared with those with AA genotype, and HRs(95% CI) for PFS and OS were 0.44(0.34-0.78) and 0.51(0.39-0.82), respectively.

***Conclusions:*** Our study suggests rs2296147 CT+TT and rs2094258 AG+GG genotypes contribute to the better survival of NSCLC. Our study provides significant information on role of prognostic value of XPG SNPs, and detecting of XPG could be used as predictive markers toward individualizing NSCLC treatment strategies.

## INTRODUCTION

The prognosis of NSCLC is poor, which was mainly due to the later diagnosis of this cancer in almost 40% of the NSCLC patients.^1^ It is estimated that the 5-years survival rate for NSCLC is always less than 15%,^2^ and NSCLC is reported to be the most fatal cancer in China in the recent 10 years.^1^ Platinum-based doublets chemotherapy is presently the standard first-line chemotherapy in advanced NSCLC patients. However, the response rate to platinum-based regimen of advanced NSCLC was less than 30%, which is lower than that for ovarian cancer, esophageal cancer and head and neck cancer.^2^ The median survival was about 7-9 months.^3^^,^^4^ Therefore, it is efficacy to select the patients who would have better response to chemotherapy to receive treatment, which could improve the efficacy of chemotherapy in advanced NSCLC.

Repair of DNA damage is a complex process, which is conducted by an array of DNA repair pathways. NER is a crucial DNA repair mechanism by which cells remove DNA lesions caused by UV radiation and some chemical agents.^5^ It is reported that a defective NER pathway can lead to xeroderma pigmentosum (XP) in human, which is mainly characterized by extreme UV-sensitivity and a high genetic predisposition to sunlight-induced skin cancers following a recessive model.^6^ Several XP complementation groups, XPA to XPG, have been identified, which represent the rate-limiting proteins in the NER mechanism.^7^

The xeroderma pigmentosum group G (XPG) gene encodes an 1186-amino acid structure-specific endonuclease, known as XPG gene, which is an indispensable component of NER and belongs to the flap structure-specific endonuclease 1 (FEN1) family.^5^ This protein also has a role in other cellular processes, which is involved in the RNA polymerase II transcription and transcription-coupled DNA repair.^8^^.^^9^ Previous experimental studies suggested that defective XPG gene polymorphisms have a vital role in the development of cancer, mainly due to DNA repair defects, genomic instability and failure of gene transcription modulation.^10^ The epidemiology studies showed polymorphisms in XPG were associated with several cancers.^11^^,^^12^ However, there were only two studies conducted in Chinese population about the association three SNPs of XPG and prognosis of advanced NSCLC receiving platinum-based chemotherapy.^13^^,^^14^ Therefore, we prospectively evaluated six SNPs of the XPG involved in the DNA repair pathway and investigate whether the six SNPs could predict the response to platinum-based chemotherapy in advanced Chinese NSCLC patients.

## METHODOLOGY

***Patients*** *: *From Nov. 2006 to Dec. 2007, 433 patients with advanced NSCLC were recruited from the First Affiliated Hospital in Xinxiang Medical College and the First Affiliated Hospital of Nanchang University in China. The eligible 433 patients were those with histologically or cytologically confirmed NSCLC, and received platinum-based chemotherapy were included in our study. The demographic and clinical data were collected from medical recorded by doctors and nurses, including sex, age, histological stage, ECOG performance status, histology and disease stage. Patients who had a history of tumor, or an already cured tumor, previous chemotherapy, radiotherapy or surgery were excluded. All the patients were followed up every two months by telephone until Nov. 2011.

All the patients in our study were treated with first-line platinum-based chemotherapy. The chemotherapeutic regimens included Vinorelbine and Cisplatin/ carboplatin; Gemicitabine and Cisplatin/ carboplatin; Taxol and Cisplatin/ carboplatin; and Docetaxel and cisplatin. Every three weeks for maximum of six cycles were used for all patients. The treatment would be ceased when disease progression or unacceptable toxicity presented. When patients presented significant 3/4 non-haematology and haematology toxicity, the dosage of treatment regimens would be decreased by 25%.

***SNP selection and genotyping:*** Genomic DNA was extracted from the buffy coat fraction of each blood sample by using a Qiagen Blood Kit (Qiagen, Chastworth, CA) according to the manufacturer’s protocol instructions. The potentially SNPs of XPG of interest were selected from NCBI dbSNP database (http://www.ncbi.nlm.nih.gov/) and SNPinfo (http://snpinfo.niehs.nih.gov/). SNPs located at two ends of XPG gene, and with the minor allele frequency (MAF) more than 5% of Chinese people, and influencing the microRNA binding sites activity. Finally, six SNPs were included in our study, including rs2296147, rs751402, rs873601, rs4150375 and rs17655 as well as rs2094258. Primers and probes were designed by primer premier software (Table-I). All the six SNPs were genotyped using the Taqman real-time PCR method with a 7900 HT sequence detector system (Applied Biosystems, Foster City, CA). To ensure high genotyping accuracy, four duplicated positive controls and four negative controls (no DNA) were used in each of 384-well plates. Approximately 10% of the samples were repeatedly genotyped, and the results were 100% concordant.

**Table-I:** Primers for genotyping XPG

*SNP*	*Primer sequence (5'-3')*	*Probe sequence (5'-3')*
rs2296147	AGCTGTCACCGCCTCCC	CGGCCATTCTCTGGACC
rs751402	GGGCTTCCAGAACTCACT	GTGTCTGTAATCGCCCTAC
rs873601	CTGGTATGAGCCCATCTA	AGTGACAAGCCTGTAGCC
rs4150375	CAATACCTTTGAGAGGGCAAGTTCA	CTCGTGATCCRCCC
rs17655	TTACGTCTTTGCGACAAATTCATT	CATTAAAGATGAACTTTCAGCAT
rs2094258	AGCCTCGCCTTTGCCGAT	CTTCTGACCCATGCCCACC

***Statistical analysis:*** Overall survival (OS) was considered as the primary end point, and it was calculated as the time from initial diagnosis to the data of death from any cause or last follow-up. Progression-free survival (PFS) was used as the secondary outcome, which was calculated from initial diagnosis to the time of progression, death without progression or last follow-up. Survival distributions were estimated by using the Kaplan-Meier method and assessed using the log-rank test. Cox proportional hazards model was used to estimate the adjusted hazard ratios (HR) with 95% CI of six SNPs of XPG for prognosis of NSCLC after adjusting for potential risk factors. Wild genotype was severed was regarded as a reference group, heterozygous variant and homozygous variant were combined to analyzed by adjusted hazard ratios (HR) with 95% CI due to the limited number of minor homozygotes. Statistical significance of all the tests was defined as two-sided with p<0.05.

## RESULTS

***Patient characteristics and clinical outcomes:*** From Nov. 2006 to Dec. 2007, patients with advanced stage NSCLC were enrolled in our study. The clinical characteristics of 433 patients were summarized in our study (Table-II). The median age of included patients was 61.4(32.5-78.7) years, and 65.7% of the patients were males. Most of the patients presented at disease stage of IV (67.4%). Most patients had histological type of adenocarcinoma (73.2%). 

In order to compare the differences between various clinical characteristics for survival of NSCLC, we compared the OS and PFS in different sex, age, ECOG performance, histology and disease stage, and we did not find significant difference in OS and PFS among patients with different characteristics.

**Table-II:** Demographic and clinical Characteristics among NSCLC patients

*Patients characteristics*	*Patients(N=433)*	*%*
*Median age (years)*	61.4(32.5-78.7)	
≤70	179	41.3
>70	254	58.7
**Sex**		
Male	284	65.7
Female	149	34.3
*ECOG performance status*		
0-1	404	93.4
≥2	29	6.6
*Histology*		
Adenocarcinoma	317	73.2
Squamous cell carcinoma	72	16.6
Large cell carcinoma	25	5.8
Other	19	4.4
*Disease stage*		
III(A/B)	141	32.6
IV	292	67.4

**Table-III:** Univariate analysis of SNPs in relation to PFS and OS among NSCLC patients

*SNP*	*Genotype*	*Cases N=433*	*PFS*					*OS*				
			*Events N=320*	*Median (month)*	*Log-rank*	*HR(95%CI)* ^1^	*P value*	*Events N=286*	*Median*	*Log-rank*	*HR(95%CI)* ^1^	*P value*
									*(month)*			
rs2296147	CC	224	187	12.3				164	19.5			
	CT+TT	209	133	17.5	<0.05	0.73(0.51-0.97)	<0.05	122	26.8	<0.05	0.66(0.48-0.99)	<0.05
rs751402	CC	245	189	14.2				168	23.5			
	CT+TT	188	131	15.6	0.43	0.91(0.67-1.23)	0.47	118	23.7	0.58	0.91(0.57-1.25)	0.61
rs873601	AA	168	140	12.2				121	20.5			
	AG+GG	265	180	17.4	0.07	0.77(0.61-1.04)	0.06	165	25.6	0.07	0.71(0.561-1.12)	0.06
rs4150375	AA	297	219	15.3				191	24.4			
	AG+GG	136	101	14.6	0.86	1.01(0.73-1.42)	0.94	95	23.1	0.74	1.09(0.79-1.57)	0.85
rs17655	GG	209	157	13.5				140	23.5			
	GC+CC	224	163	14.6	0.75	0.95(0.72-1.32)	0.81	146	25.2	0.17	0.97(0.71-1.34)	0.11
rs2094258	AA	158	140	12.5				128	19.2			
	AG+GG	275	180	18.4	<0.05	0.44(0.34-0.78)	<0.05	158	27.3	<0.05	0.51(0.39-0.82)	<0.05

1. Adjusting sex, age, histological stage, ECOG performance status, histology, disease stage and chemotherapy regimens.

**Fig.1:**
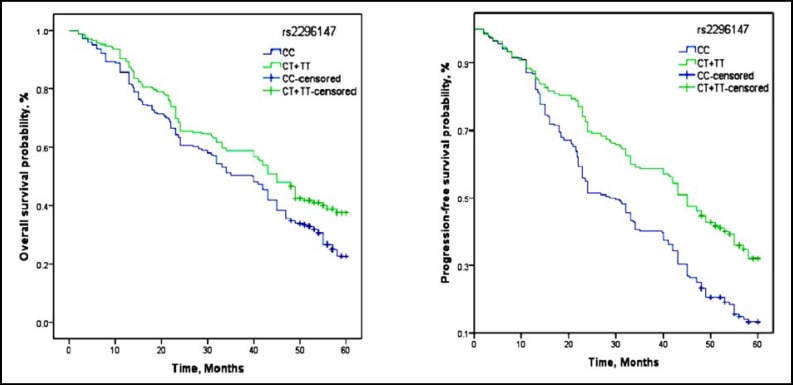
Kaplan-Meier estimates of PFS and OS with rs2296147 in advanced NSCLC patients

**Fig.2:**
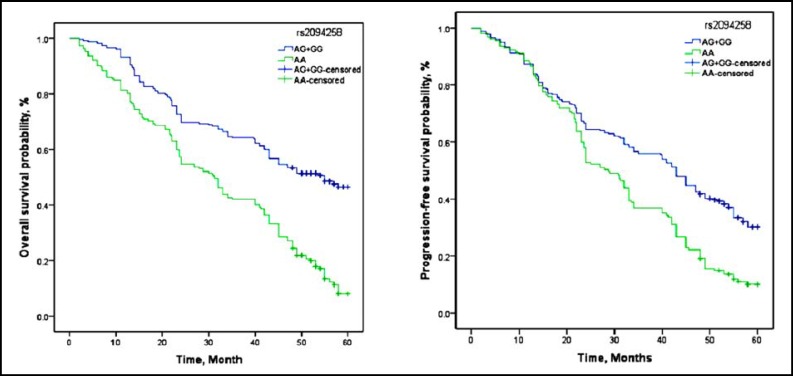
Kaplan-Meier estimates of PFS and OS with rs2094258 in advanced NSCLC patients

***Effect of six SNPs on PFS and OS:*** The genotype frequencies of six SNPs of XPG are shown in Table-III. The genotype frequencies of six SNPs of XPG among 433 patients were according to Hardy-Weinberg equilibrium (data not shown). 

In our study, patients with rs2296147 CT+TT genotype and rs2094258 AG+GG genotype showed a significantly longer median PFS (17.5 months and 18.4 months) and OS (26.8 months and 27.3 months) than wild genotype, and also showed significantly different in PFS and OS compared to CC genotype by log-rank test (Table-III, Fig.1 and Fig.2). In the Cox proportional hazards model after adjusting for potential confounding factors, we found the hazard ratio (HR) for progressive in patients with rs2296147 CT+TT genotype and rs2094258 AG+GG genotype was 0.73(0.51-0.97) and 0.44(0.34-0.78), respectively, when compared genotype as a reference variable. Moreover, HRs(95% CI) of OS were 0.66(0.48-0.99) and 0.51(0.39-0.82), respectively.

The analysis of rs751402, rs873601, rs4150375 and rs17655 did not show significant effect on the PFS and OS of advanced NSCLC patients.

## DISCUSSION

In the current study, we found the rs2296147 CT+TT and rs2094258 AG+GG genotypes were associated with decreased risk of progression and death. Our study was the first study to investigate the effect of six SNPs of XPG on the clinical outcome of advanced NSCLC treated with platinum-based chemotherapy, and our study indicated the down regulation of XPG activity leads to enhanced survival time of advanced NSCLC with platinum-based chemotherapy. This is possibly the function of XPG in the NER pathway which repairs DNA damage caused by platinum-based chemotherapy.

NER pathway has the function of repairing bulky, helix-distorting adducts, of which formed by cisplatin and its analogs.^15^^,^^16^ The cisplatin-DNA adducts could be removed by platinum-resistant cells during the NER pathway and thus escape apoptosis.

XPG is responsible for a 1186 amino acid structure-specific endonuclease, the activity of which is essential for the two incision steps in NER. In human cells, XPG catalyzes an incision approximately 5 nucleotides 3’ to the site of damage and is also involved non-enzymatically in the subsequent 5' incision.^17^ An increasing number of previous studies have indicated the role of NER pathway in cellular response to platinum chemotherapy, such as enhanced NER activity to the resistance of platinum agents and diminished NER activity to the sensitivity of platinum agents. Several studies indicated XPG may have effect on the several cancers with chemotherapy.^18^^,^^19^ A previous study conducted in Western China with 83 colorectal cancer patients showed XPG +25AA genotype was significantly higher than that of other genotypes, and patients carrying -763A/+25G haplotype had a higher risk of non-response to oxaliplatin chemotherapy when compared with those carrying -763G/+25A haplotype (OR=2.67).^18^ Another study conducted in Northern China indicated patients with wide-type rs17655 GG genotype were observed with a longer progression-free survival after oxaliplatin-based adjuvant chemotherapy, with a hazard ratio of 1.69.^19^

There were only two studies which indicated that XPG promoter polymorphisms contribute to clinical outcome of NSCLC patients receiving platinum-based chemotherapy.^13^^,^^14^ One study conducted in China investigated three SNPs in the XPG promoter region indicated rs751402 AA genotype increased the chemotherapy response to advanced NSCLC, and no significant association was found between rs2094258 and rs2296147 polymorphisms and treatment response.^13^ Another study also conducted in China indicated rs17655 in the NER pathway was correlated with toxicity treated with chemotherapy in advanced NSCLC.^14^ In our study, we found rs2296147 and rs2094258 were associated with PSF and OS in NSCLC, which was different from the results of previous studies. The possible discrepancy of the results of XPG polymorphisms in clinical outcome of NSCLC receiving platinum-based chemotherapy may be due to different backgrounds of cases, sample size, sample size and etc.

The finding of our study has indicated there was association between SNPs of XPG and survival in advanced NSCLC patients receiving platinum-based chemotherapy, and suggesting rs2296147 CT+TT and rs2094258 AG+GG contribute to the better survival of NSCLC. Our study provides significant information on role of prognostic value of XPG SNPs, and detecting of XPG could be used as predictive markers toward individualizing NSCLC treatment strategies.
